# STING Signaling and Sterile Inflammation

**DOI:** 10.3389/fimmu.2021.753789

**Published:** 2021-10-01

**Authors:** Isabelle Couillin, Nicolas Riteau

**Affiliations:** Experimental and Molecular Immunology and Neurogenetics Laboratory (INEM), Centre National de la Recherche Scientifique (CNRS), UMR7355 and University of Orleans, Orleans, France

**Keywords:** STING, cGAS, self-DNA, sterile inflammation, autophagy, type I IFN

## Abstract

Innate immunity is regulated by a broad set of evolutionary conserved receptors to finely probe the local environment and maintain host integrity. Besides pathogen recognition through conserved motifs, several of these receptors also sense aberrant or misplaced self-molecules as a sign of perturbed homeostasis. Among them, self-nucleic acid sensing by the cyclic GMP-AMP synthase (cGAS)/stimulator of interferon genes (STING) pathway alerts on the presence of both exogenous and endogenous DNA in the cytoplasm. We review recent literature demonstrating that self-nucleic acid detection through the STING pathway is central to numerous processes, from cell physiology to sterile injury, auto-immunity and cancer. We address the role of STING in autoimmune diseases linked to dysfunctional DNAse or related to mutations in DNA sensing pathways. We expose the role of the cGAS/STING pathway in inflammatory diseases, neurodegenerative conditions and cancer. Connections between STING in various cell processes including autophagy and cell death are developed. Finally, we review proposed mechanisms to explain the sources of cytoplasmic DNA.

**Graphical Abstract:**
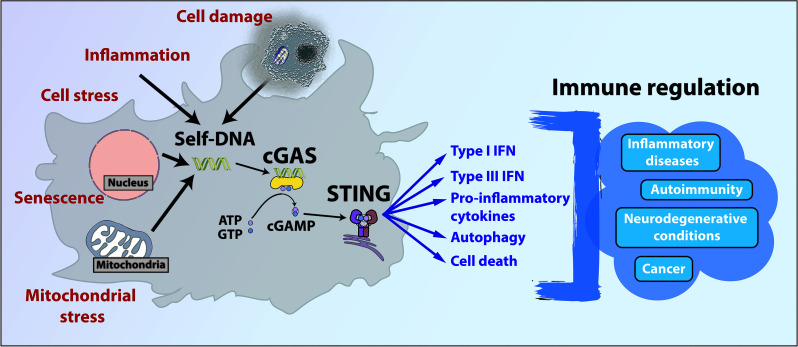
Illustration of the diversity of processes triggering self-DNA mediated cGAS/STING activation and subsequent pathways involved in the immune regulation of various conditions.

## Introduction

Supported by Charles Janeway’s central model of innate immunity based on pathogen recognition through conserved motifs (1), host detection of pathogen-derived nucleic acids by pathogen recognition receptors (PRRs) is a common and effective strategy to sense invading microorganisms and initiate innate and adaptive immune responses (2). PRRs are also involved in context-dependent recognition of self-nucleic acids, either nuclear DNA (nDNA) or mitochondrial DNA (mtDNA) (3–6). This is in line with Polly Matzinger’s model proposing that in numerous instances what ultimately matters for a host is to detect danger independently of its external or internal origin to mount an immune response. Cell stress or damage results in the production and/or the release of host-derived danger signals (7). Apart from mitosis, cell DNA is confined within the nucleus or mitochondria. DNA in the cytoplasm, from microbial origin (e.g. following viral or bacterial infection) or from the host own genetic material, informs of a potentially deleterious situation and the latter constitutes an endogenous danger signal.

Among PRRs involved in self-nucleic acid recognition, an increasing interest has emerged for STimulator of Interferon Genes (STING), also known as TMEM173, MPYS or MITA (6, 8–10). STING is an endoplasmic reticulum (ER)-located PRR, which does not directly bind to DNA. Its activating ligands are cyclic dinucleotides (CDNs). CDNs are produced as second messengers by microorganisms (11, 12) or synthesized by the enzyme cyclic GMP-AMP synthase (cGAS) in response to binding either host- or pathogen-derived cytosolic double-stranded (ds)DNA (6, 13–15). While displaying no apparent DNA sequence specificity (16), cGAS activity is limited by spatial distribution of its substrate as well as its degradation by host DNases. cGAS binds to nDNA (17) or mtDNA (18) that can be continuous, fragmented or supercoiled (17). Crystal structure analysis of mouse cGAS revealed that it functions as a dimer, with each protein catalytic domain binding to an 18 base pairs (bp) double-stranded (ds) DNA at two different sites, forming a 2:2 complex (19, 20). While initially described as a cytoplasmic protein, a recent study found that cGAS binds to the inner leaflet of the plasma membrane to regulate its activity and prevent overactivation from genotoxic stress (21). Other studies proposed that cGAS resides predominantly in the nucleus of various mouse and human cell lines, as well as human peripheral blood mononuclear cells (PBMC)-derived macrophages and dendritic cells (22, 23). However, its baseline activation in the nucleus is prevented by poorly accessible nucleosomal state of nDNA (20, 22, 24). Thus, cGAS cellular localization appears unclear and additional studies are required.

**Figure 1:**
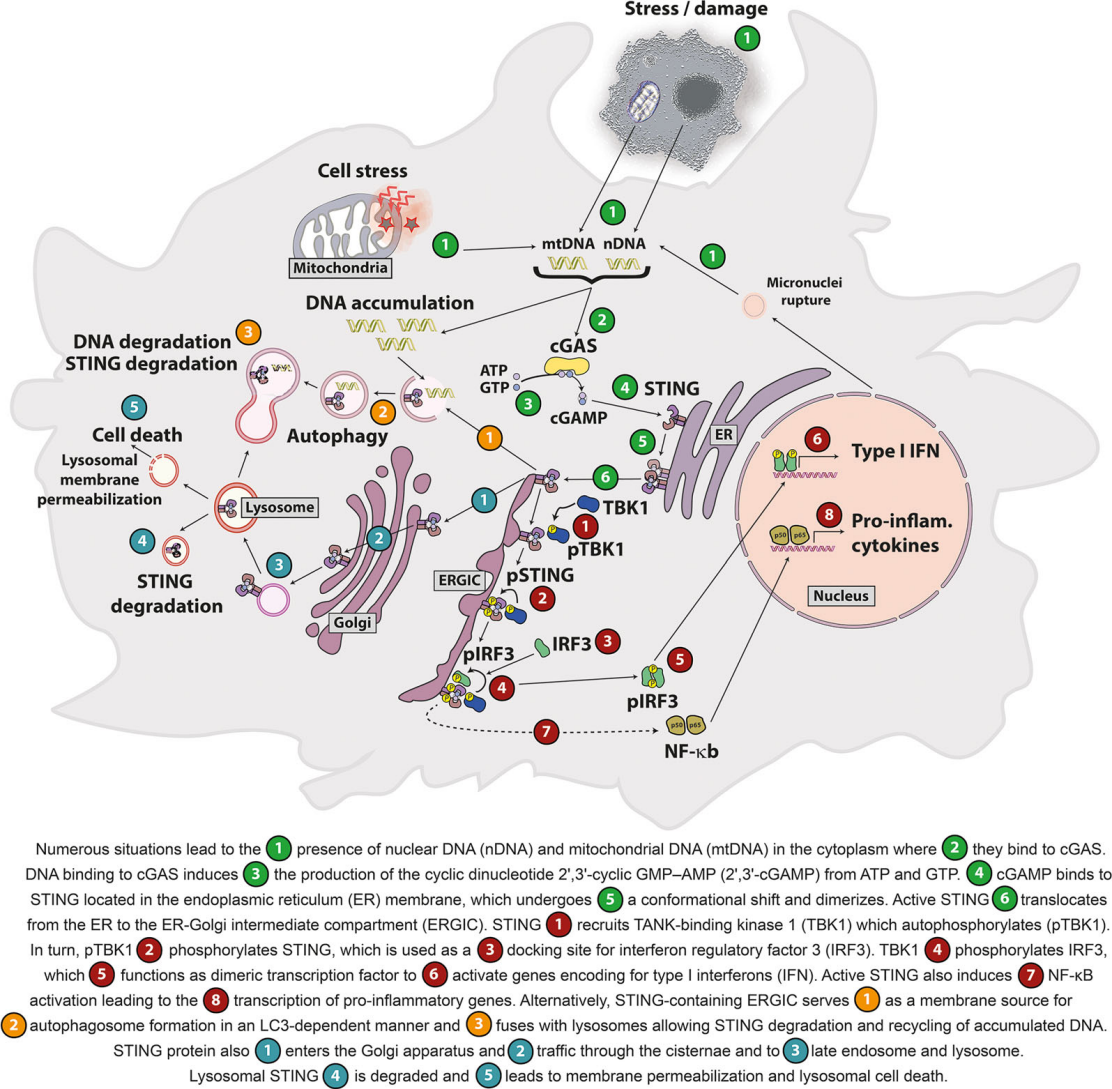
Self-DNA-mediated activation of the cGAS/STING pathway.

DNA binding to cGAS triggers ATP and GTP conversion into cyclic guanosine monophosphate–adenosine monophosphate (cGAMP). cGAMP is the canonical CDN that binds and activates STING (**Figure 1**). cGAMP binding to STING elicits a conformational shift and its dimerization as well as its translocation to the ER-Golgi intermediate compartment (ERGIC). STING dimers recruit TANK-binding kinase 1 (TBK1), which phosphorylates STING on Ser366 to serve as a docking site for interferon regulatory factor 3 (IRF3) and its phosphorylation by TBK1. STING also leads to nuclear factor κB (NF-κB) activation. IRF3 and NF-κB transcription factors induce the production of type I interferons (IFNs) and other cytokines involved in host immune responses (6, 8, 25, 26). Interestingly, recent studies showed that STING stimulation can also lead to type III IFN production (also known as IL-28/IL-29 or IFN-λ) (27–29). Besides its roles in the regulation of gene expression, STING induces non-canonical autophagy through its direct interaction with the microtubule-associated protein light chain 3 (LC3), a key initiator of autophagy that cycles between the nucleus and cytoplasm (25, 30–32). Importantly, STING-induced autophagy mediates the clearance of cytosolic DNA (30). STING also regulates various cell death processes including lysosomal cell death (33), apoptosis (34) and necroptosis (35). Of note, in addition to cGAS, other cytosolic receptors (e.g. DDX41, IFI16) can sense DNA or CDNs and activate STING (36, 37). The cGAS/STING pathway is important to control viral and bacterial pathogen infection (38, 39) as well as for immune surveillance (40, 41). STING is ubiquitously expressed in a variety of tissues including lungs, liver, kidney, heart and spleen (8). STING is expressed in both innate and adaptive immune cells (e.g. macrophages (8), dendritic cells (8), natural killer cells (42), CD4^+^ and CD8^+^ T lymphocytes (43) and B lymphocytes (44). It is also expressed by nonhematopoietic-derived cells including endothelial cells (45), epithelial cells (46) and neurons (47).

Here, we detailed known mechanisms to explain DNA access to the cytoplasm, briefly review STING allelic variants and then focus on STING biology in the context of self-DNA sensing associated with autoimmunity, cancer or sterile inflammatory settings.

## DNA Access to Cytoplasm

**Figure 2:**
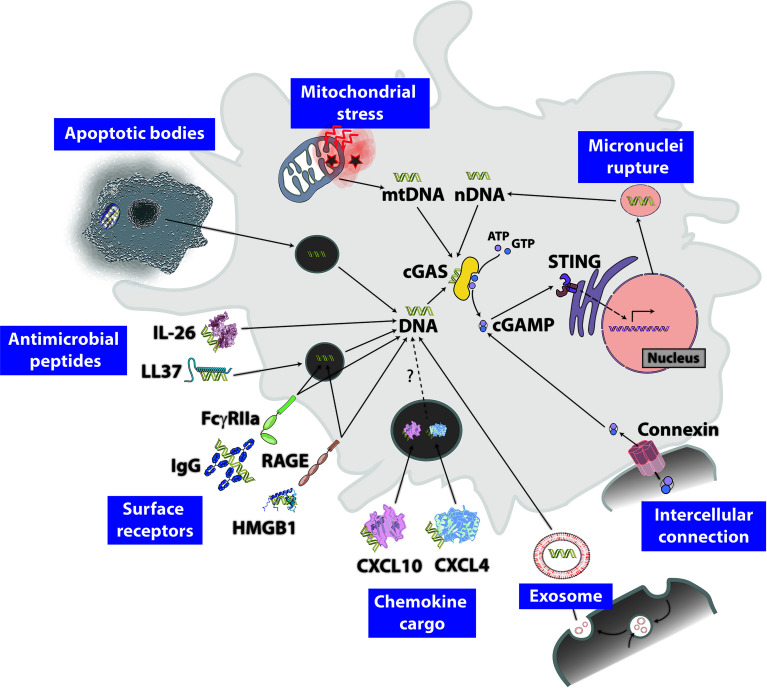
Proposed mechanism for self-DNA access to the cytoplasm. Mitochondrial stress-induced membrane permeability with mitochondrial DNA (mtDNA) leakage and chromosomal instability/senescence lead to micronucleus formation from incomplete segregation of chromatin and micronucleus rupture induces cytosolic nuclear DNA (nDNA). Multiples pathways lead to self-DNA internalization within endosomal structures and we believe that endosomal pathway perturbation leads to cytosolic delivery of DNA and cGAS/STING pathway activation. Apoptotic bodies engulfment may enable self-DNA delivery in the cytoplasm. The antimicrobial peptide LL37 and IL-26 bind to genomic DNA and induce its translocation within endosomes and/or in the cytoplasm. CXCL4- and CXCL10-DNA complexes as well as cell surface receptors FcγRIIa or receptor for advanced glycation end products (RAGE) bind to IgG- or HMGB1-bound DNA, respectively, and may lead to cGAS activation upon cytosolic DNA release. DNA-containing exosomes constitute another source of cGAS activation. Finally, cGAMP enters the cell from neighboring ones through gap junctions to directly activate STING.

Host DNA, which resides within the nucleus or mitochondria, may be released into the cytoplasm following numerous processes, including genomic DNA instability, mitochondrial stress and endosomal/lysosomal rupture (**Figure 2**). For instance, nDNA-containing micronuclei rupture (17, 48) and mitochondrial stress (49–52) lead to DNA release in the cytoplasm and cGAS activation. MtDNA stress elicited by mitochondrial transcription factor A (TFAM) deficiency induces its release into the cytosol and activates the cGAS/STING pathway leading to type I IFN-dependent interferon stimulated genes (ISGs) expression and increased antiviral capacity (26). DsDNA from dying cells stimulates STING pathway in surrounding cells, suggesting that extrinsic phagocytic DNA improperly processed within lysosomes access the cytosol (53, 54). The mechanisms by which self-DNA from neighboring cells becomes accessible for intracellular DNA sensors remain uncertain. Several context-dependent pathways have been reported, such as IgG- or HMGB1-bound DNA internalization following interaction with FcγRIIa or receptor for advanced glycation end products (RAGE), respectively (55). The antimicrobial peptide LL37 was shown to transport extracellular DNA into the cytoplasm of human primary monocytes triggering STING activation (56). IL-10-family member IL-26 binds to genomic, mtDNA or neutrophil extracellular traps (NETS) DNA and traffic them into the cytosol of human myeloid cells activating STING (57). Radiotherapy-induced cytosolic dsDNA accumulation in cancer cells activates the cGAS/STING pathway to promote type I IFN production-dependent protective effect, notably by recruiting BATF3-dependent DCs (58). It was shown that exosomes-containing DNA from cancer cells can be transferred to the cytoplasm of DCs (58).

Besides chemotactic properties following their binding to chemokine receptors (CXCR), several chemokines display other functions. In particular, CXCL4 and CXCL10 have been shown to activate endosomal TLR9. CXCL4 binds to self and foreign DNA to form liquid crystalline complexes amplifying TLR9-mediated IFN-α production in systemic sclerosis (59). Neutrophil-derived CXCL10 binds to commensal skin microbiota DNA and triggers TLR9/type I IFN-dependent innate repair responses in injured skin (60). It remains to be determined whether CXCL4- or CXCL10-DNA complexes can activate intracellular sensors such as cGAS.

cGAMP transfer from producing cells to neighboring ones through gap junctions promotes STING activation and antiviral immunity independently of type I IFN signaling (61). cGAS-derived cGAMP in tumor cells diffuses to neighboring non-cancerous cells through gap-junction channels to activate STING, contributing to the recruitment of protective tumor-infiltrating immune cells such as NK cells (62).

## STING Allelic Variants

There are several STING allelic variants in the general population (63). The most commons are R232H and R71H-G230A-R293Q (HAQ). Their prevalence varies among ethnic populations. On average, about 15% and 20% of worldwide population carries at least one copy of R232H or HAQ variants, respectively (63–65). Alterations in STING biology induced by these point mutations are not fully clear and apparent discrepancies have been reported. Using ectopic overexpression systems, human HAQ variant exhibited a decreased response to exogenous CDNs (63) while its response to 2’3’cGAMP was either normal (63) or absent (64). STING R232H showed reduced responsiveness to most exogenous CDNs although its response to 2’3’cGAMP was relatively normal (63, 64) or reduced (66). Interestingly, it was shown that R293Q STING variant displays protective effects against obesity-associated cardiovascular disease (67) and tobacco-induced aging-associated diseases (68). In contrast, gain-of-function point mutations in the gene encoding STING (e.g. V155M) are responsible for STING-associated vasculopathy with onset in infancy (SAVI), a rare inflammatory and autoimmune condition (69, 70).

## STING and Autoimmune or Autoinflammatory Diseases

### STING-Associated Vasculopathy With Onset in Infancy

In 2014, it was discovered that point gain-of-function mutations in *TMEM173* (i.e. N154S, V155M, and V147L) from six unrelated children cause SAVI (69). SAVI is an autoinflammatory disease with early-onset systemic inflammation, cutaneous vasculopathy and interstitial lung disease (69, 71). Children PBMCs display constitutive STING activation, leading to higher transcription baseline for *IFNB1*, *IL6* and *TNF* as well as ISGs such as CXCL10 as compared to PBMCs from healthy controls. In contrast, baseline and cGAMP-induced transcription levels of *IFNA4*, *IFNG* and *IL1B* were similar between patients and controls (69). The same year, V155M point mutation in STING was also found in patients with familial lupus-like phenotypes (70). STING mutant spontaneously localizes in the Golgi of patient fibroblasts and is constitutively active in the absence of exogenous 2′3′-cGAMP *in vitro* (70).

Two STING knock-in mouse strains corresponding to mutations found in SAVI patients have been generated. Heterozygous V154M STING (equivalent to V155M in patients) and heterozygous STING N153S (equivalent to N154S in patients) mouse strains both display severe combined immunodeficiency disease (SCID) phenotype and thymocytes impairment at early stages (72–74). Interestingly, STING N153S-associated lung disease is T lymphocyte-dependent but does not require IRF3/IRF7 nor type I IFN signaling (75). STING-associated vasculopathy also develops independently of IRF3 in mice (73).

### Bloom Syndrome

Bloom syndrome is a rare autosomal recessive genetic disorder caused by mutations in the BLM gene encoding BLM RecQ–like helicase (76), which maintains DNA stability during cell replication. BLM protein deficiency or lack of protein activity leads to increased mutations. Bloom syndrome is characterized by short stature, cancer predisposition, genomic instability and accumulation of micronuclei. A recent study showed that BLM-deficient fibroblasts display constitutive ISGs up-regulation in a cGAS/STING/IRF3-dependent mechanism (77). Further investigation are required to determine the exact contribution of the STING pathway in Bloom syndrome.

### Multiple Sclerosis

Multiple sclerosis (MS) is a demyelinating condition that can affect both the brain and spinal cord. It was shown that STING activation attenuates experimental autoimmune encephalitis (EAE) utilized as an MS model by attenuating effector T cell infiltration and inducing a dominant T regulatory (Treg) response (78). Systemic treatments with DNA nanoparticles or CDNs activate the STING/type I IFN pathway enhancing indoleamine 2,3 dioxygenase (IDO) enzyme activity in dendritic cells promoting Treg cells (78). Another study showed that FDA-approved antiviral drug ganciclovir (GCV) induces a STING-dependent type I IFN response inhibiting inflammation in cultured myeloid cells and in EAE model (79). Together, STING pathway appears to be an important regulator of microglial reactivity and neuroinflammation with possible beneficial therapeutic effects for MS patients.

### Type I Diabetes

Type 1 diabetes (T1D), sometimes referred to as juvenile diabetes, is caused by the destruction of pancreatic beta cells resulting in insufficient amount of insulin and hence elevated blood sugar levels. High glucose environment increases ROS production, triggering mitochondrial stress and mtDNA release in retinal cells and activating the cGAS/STING pathway leading to IRF3 activation *via* ERK1/2-Akt-tuberin - mechanistic target of rapamycin (mTOR) dependent pathways (49). Treatments with DNA nanoparticles or cGAMP attenuate type I diabetes progression in non-obese diabetic (NOD) female mice by inducing type I IFN-dependent IDO activity which promotes Treg cell function and therefore limits autoimmunity (80). It was further shown that TBK1 in monocytes from type 1 diabetes patients is important for IFN-α production in response to CpG DNA stimulation (81).

### Dysfunctional DNase and Autoimmune Diseases

An expected consequence of self-DNA-triggered immunity is tolerance breakdown and autoimmunity (82, 83). Host cytoplasmic DNA sensing is typically prevented since it is usually restricted to the nucleus and mitochondria. However, multiple situations lead to the presence of misplaced DNA and autoimmunity can result from failure to properly dispose it through DNAse activity. Besides cell extrinsic DNAse I, mammalian cells express the endonuclease DNase II and the exonuclease TREX1/DNase III located within lysosomes and in the cytosol in an ER tail-anchored manner, respectively. In human, loss-of-function mutations in TREX1 trigger autoimmune diseases, such as Aicardi–Goutières syndrome (AGS), systemic lupus erythematosus (SLE), familial chilblain lupus (FCL), and retinal vasculopathy with cerebral leukodystrophy (RVCL) (84, 85). Recent data suggest that STING pathway may be involved in these autoimmune diseases linked to dysregulated nucleases activity (84).

### Aicardi-Goutières Syndrome

Aicardi-Goutières syndrome (AGS) is a neuroinflammatory autoimmune disease triggered by mutations in genes encoding nucleotide-processing proteins. *Trex1*-deficient mice succumb from systemic inflammation during early adulthood (86, 87) whereas *DNase II*-deficient mice are embryonic lethal (88). Interestingly, lethality observed in both *Trex1* or *Dnase II*-deficient mice is rescued by crossing these mice to either *cGas* or *Sting*-deficient mice (54, 89). It was shown that STING-mediated type I IFN production in *Trex1*-deficient mice occurs first in nonhematopoietic cells, which trigger T and B lymphocytes-mediated inflammation and autoantibody production (90).

RNase H2 is essential to remove ribonucleotides incorporated in genomic DNA during replication. Defective RNase H2 leads to AGS by promoting self-nucleic acid accumulation leading to chronic type I IFN production. A knock-in mouse strain containing an RNase H2 AGS mutation (G37S) shows perinatal lethality. This phenotype is partially rescued when these mice are crossed with *Sting*-deficient mice (91), confirming that STING signaling pathway is involved in AGS. Using *in vitro* RnaseH2a G37S/G37S mouse embryonic fibroblast (MEF) cell cultures, the authors confirmed that the cGAS/STING pathway triggers type I IFN and ISGs upregulation (91). The exact mechanism by which defective RNase H2 leads to cGAS activation remains to be determined.

### Systemic Lupus Erythematosus

Systemic lupus erythematosus (SLE) development is classically linked to endosomal toll like receptor (TLR) 7 and TLR9 nucleic acid sensors engagement and the role of the cGAS/STING pathway remains elusive. PBMCs from SLE patients express high levels of cGAS (92). It was recently shown that in contrast to healthy donors, most monocytes from lupus patients produce IFN-α following 2’3’-cGAMP stimulation and the frequency of IFN-α producing monocytes positively correlates with SLE disease activity (93). Interestingly, mTOR inhibition suppressed STING upregulation and IFN-α production in lupus monocytes (93). In mice, *Fas*-deficient lupus-prone MRL/Mp-lpr/lpr mice display systemic autoimmunity, massive lymphadenopathy, arthritis and immune complex glomerulonephrosis starting at about three months of age. Crossing these lupus-prone mice with *Sting*-deficient mice significantly increases autoimmunity and shortens lifespans (94). The authors further show that STING-mediated protection is IRF3-independent (94). Furthermore, TLR-dependent systemic inflammation following 2,6,10,14-tetramethylpentadecane (TMPD) stimulation is exacerbated by STING deficiency which results in increased levels of pro-inflammatory cytokines and elevated numbers of myeloid cells (94).

### Familial Chilblain Lupus

Familial chilblain lupus is a rare autosomal dominant form of cutaneous lupus erythematosus occurring mainly in young children, due to mutations in the *TREX1* gene as well as in mutations within *SAMHD1* or *TMEM173* in rare cases (95, 96). In particular, G166E heterozygous mutation in *TMEM173* was found in five patients with familial chilblain lupus resulting in constitutive type I IFN production, for which the Janus kinase (JAK) inhibitor tofacitinib showed promising results (95).

## CGAS/STING in Inflammatory Diseases

### Fibrosis

STING pathway has been linked to the regulation of fibrotic processes in various tissues. Idiopathic pulmonary fibrosis (IPF) is characterized by progressive lung scarring punctuated by life-threatening acute exacerbations causing shorter life expectancy and a high mortality rate (97–99). It is believed that the physiopathology relies on repetitive local micro-injuries leading to DNA damage, cell death and finally to an aberrant repair with deposition of extracellular matrix components and fibrosis (97–100). Using the classical murine model of human IPF by airway exposure to bleomycin (BLM), we published recently that STING deficiency leads to increased lung fibrosis in an unexpected type I IFN-independent manner indicating that STING plays a protective role in limiting experimental lung fibrosis (101). In line with our findings, a recent study in IPF patients showed that STING expression in PBMC decreases during acute exacerbation. STING protein levels post-treatment increased in patients showing clinical improvement but remained low in patients displaying clinical deterioration, strongly suggesting a benefic role of STING in IPF (102). Of note, elevated plasma mtDNA copy numbers in IPF patients predict death (103). A protective role of STING against fibrosis has also been shown is a model of chronic pancreatitis (104). Authors showed that STING deficiency promotes Th17 polarization and IL-17A production therefore pancreatic inflammation and fibrosis (104).

On the other hand, Susztak et al. showed that patients suffering from chronic kidney disease (CKD) display defective mitochondrial integrity and further linked mtDNA release, STING activation and renal fibrosis (50). STING expression is upregulated in human and mouse hypertrophic hearts and STING deficiency leads to decreased inflammation and fibrosis in an ER stress-associated process (105). Inflammation-driven liver fibrosis upon carbon tetrachloride (CCl4) administration is a well-established model of chronic liver disease. It was shown that STING/IRF3 pathway mediates hepatocyte death and fibrosis independently of TLR and type I IFN signaling (106). Of note, the same group showed that STING is activated by alcohol-induced ER stress to trigger IRF3-dependent hepatocyte apoptosis, independently of type I IFN signaling (107). Chronic silica particles inhalation triggers silicosis, a lung disease with progressive interstitial fibrosis and increased risk of cancer. It was shown that airway silica exposure induces both nDNA and mtDNA release and STING-dependent type I IFN responses. *Sting*-deficient mice displayed significantly attenuated lung inflammation (108). Thus, STING contribution to fibrosis appears highly context/organ dependent and further studies are required to delineate the molecular mechanisms involved.

### Obesity-Related Diseases

Chronic sterile inflammation in obesity and related metabolic diseases such as type 2 diabetes, nonalcoholic fatty liver disease (NAFLD) and cardiovascular disease have been widely demonstrated (109). A number of studies recently showed that self-DNA sensing, either nuclear or mitochondrial, plays important roles in these pathologies. Obesity promotes mtDNA release into the cytosol of adipocytes, which leads to cGAS-STING-mediated inflammation (51). Using methionine/choline-deficient diet (MCD) or high-fat diet (HFD) as murine models of non-alcoholic steatohepatitis (NASH), it was shown that STING/IRF3 pathway promotes hepatocyte injury and dysfunction by inducing inflammation and apoptosis as well as by disturbing glucose and lipid metabolism (110). *Sting*-deficient mice display reduced HFD-induced adipose tissue inflammation and insulin resistance (111). However, the cell subset(s) involved remain(s) unclear. A recent study confirmed that STING deficiency attenuated steatosis, fibrosis, and inflammation using both MCD and HFD murine models (52). However, they pointed out that in contrast to IRF3, several studies including theirs reported that STING protein is not expressed in hepatocytes of adult humans or mice. They showed that hepatocytes from HFD-fed mice release mtDNA which activates STING in Kupffer cells leading to TNF-α and IL-6 productions and pathology (52). The role of STING in hematopoietic-derived cells in driving HFD-induced NAFLD has been confirmed by bone marrow transfer experiments (112). However, STING role in non-immune cells has also been confirmed. Adipose tissue chronic inflammation and metabolic stress in obesity induce endothelial inflammation, which plays a key role in insulin resistance. Obesity-related increase in free fatty acid induces mitochondrial damage and mtDNA release, which activates the cGAS/STING pathway leading to IRF3-dependent upregulation of ICAM-1 expression and endothelial inflammation (111).

### Myocardial Infarction

An elegant study showed that ischemic cell death and cell debris uptake by cardiac macrophages lead to cGAS/STING pathway activation and IRF3-mediated type I IFN production (113). As compared to WT relatives, myocardial infarction induction in *Sting*-deficient mice leads to decreased *Ifnb1* expression and a strong drop in *Cxcl10*, *Irf7*, and *Ifit1* expressions (113). In contrast to about 50% mortality in WT mice, *Irf3*- or type I IFN receptor (*Ifnar*)-deficient mice showed virtually complete protection, whereas *Cgas*-deficient mice displayed partial protection and *Sting*-deficient mice did not reach statistical difference (113). These data suggest that other innate immune sensors are involved in IRF3 activation after myocardial infarction.

Upon myocardial infarction, cGAS induces iNOS and CXCL10 upregulation but not pro-inflammatory mediators such as IL-1β, IL-18, TNF-α and IL-6 (114). *Cgas*-deficient mice display higher regulatory capacities, including M2-like macrophages and myofibroblasts in the region bordering the myocardial infarction. cGAS deficiency also protects against myocardial infarction-induced adverse ventricular remodeling and rupture and enhances tissue repair in the infarct region (114). Of note, it was suggested that cGAS-mediated autophagy protects the liver from ischemia-reperfusion injury independently of STING (115).

### COPD

Chronic obstructive pulmonary disease (COPD) is a major health issue primarily caused by cigarette smoke (CS) inhalation. It is characterized by chronic bronchitis and emphysema, i.e. long-term inflammation of the airways and irreversible destruction of the alveolar cell wall, respectively (116). Using an acute model of CS exposure in mice, we showed that CS increases self-DNA content in the alveolar space driving cGAS/STING-dependent neutrophilic influx and inflammatory response (117). DNAse I treatment reduces CS-induced lung inflammation by limiting deleterious effects of neutrophil extracellular traps (NETs) for instance in terms of protease expression (118). In contrast, sub-chronic CS exposure induces a reduction of STING lung expression impeding subsequent response to infection (119) and COPD patients display lower pulmonary IFN-β expression (120). Lower IFN levels might in part contribute to the poor immune response to infection developed by COPD patients during exacerbation phases.

## CGAS/STING in Neurodegenerative Disorders

Over the past few years, a number of important studies showed potential strong involvement of the cGAS/STING pathway in neuroinflammatory processes and neurodegenerative disorders. STING regulates steady-state and nerve injury-triggered nociception through its signaling in sensory neurons (121). Intrathecal injection (i.e. into the spinal canal) of STING agonists leads to robust antinociception in mice and non-human primates (NHPs) in a type I IFN-mediated signaling on peripheral nociceptors (121). *Sting* or *Ifnar*-deficient mice exhibit hypersensitivity to nociceptive stimuli and increased nociceptor excitability. The exact mechanism of STING activation at steady state and following nerve damage remains to be determined, potentially implying cell death and self-DNA sensing.

Amyotrophic lateral sclerosis (ALS) is a neurodegenerative disease of the central nervous system (CNS) causing progressive loss of muscle control and often characterized by a cytotoxic accumulation of TAR DNA-binding protein 43 (TDP-43). TDP-43 accumulation in the mitochondria induces DNA leakage and cGAS/STING pathway activation promoting inflammatory signaling and pathology (122). Using mouse models to investigate Parkinson’s disease (PD) inflammatory profile, Sliter et al. showed that inflammation in *Parkin* ^-/-^ and *Pink1* ^-/-^ mice undergoing exhaustive exercise is linked to mtDNA leakage and rescued in the absence of STING (123). Serum/glucocorticoid related kinase 1 (SGK1) is upregulated in the brains of patients with various neurodegenerative disorders such as PD and pharmacological inhibition of SGK1 limits NLRP3-inflammasome- and cGAS-STING-mediated inflammatory pathways (124). Employing PD mouse models, it was shown that the neuroprotective agent withaferin A protected against dopaminergic neuron loss in a STING-dependent manner (125).

The cGAS/STING pathway might also play a role in accelerated aging and neurodegeneration observed in Huntington’s disease (HD). Melatonin, a radical scavenger expressed by neuronal mitochondria, decreases with aging and neurodegeneration. Melatonin-deficient mice display increased mtDNA release and activation the cGAS/STING/IRF3 pathway (126). Exogenous melatonin administration in R6/2 mice as a genetic mouse model of HD alleviates cGAS/STING-mediated inflammation (126). STING pathway inhibition may offer therapeutic benefits in HD by limiting deleterious up-regulation of inflammatory and autophagy responses (127). Finally, cGAS/STING activation delayed neurodegeneration in neonatal hypoxia-ischemia in rats (128), however STING-mediated protective mechanism remains to be determined.

## CGAS/STING in Cancer

cGAS/STING pathway in cancer settings is under deep examination in both tumor cells as well as in neighboring immune and non-immune cells. Most tumors retain cGAS and STING expression (129) and cancer cell cGAS recognizing cytosolic DNA produces cGAMP inducing STING-dependent type-I IFN secretion (130). Acute STING activation is likely to exhibit type I IFN-mediated anti-tumor effect associated with cellular senescence and T lymphocyte-dependent immunity. In more advance stages, chromosomally unstable tumors become tolerant to chronic cGAS-STING signaling, downregulate downstream IFN signaling while maintaining alternative pathways that promote tumorigenesis (129). In mice, STING deficiency facilitates development of several types of tumors whereas STING stimulation favors antitumor immunity (41, 131, 132). Cytosolic DNA accumulation can result from the combined action of the endonuclease MUS81 and PARP-dependent DNA repair as shown in prostate cancer cells, leading to STING-dependent tumor rejection (133). In contrast, by generating Sting^S365A/S365A^ mutant mouse strain that precisely ablates IFN-dependent activities, Wu et al. showed that T cells in tumors undergo substantial cell death in part mediated by IFN-independent STING activities promoting tumor evasion (134).

cGAS also elicits contrasting outcomes depending on the context. Carcinoma-derived cGAMP diffuses to astrocyte through connexin 43 (Cx43) gap junctions leading to STING-dependent IFN-α production acting in paracrine manner on metastatic cells to support chemoresistance and tumor growth through STAT1 activation (135). DNA repair following double-stranded breaks by homologous recombination prevents tumorigenesis. DNA damage induces nuclear translocation of cGAS where it inhibits homologous recombination and therefore promotes tumor growth (136). A recent publication showed that efferocytosis blockade by inhibiting MerTK-dependent apoptotic tumor cell phagocytosis promotes CD8^+^ T cell-mediated anti-tumor activity. Apoptotic tumor cells clearance failure leads to secondary necrosis accompanied with danger signals release. Among them, ATP and cGAMP activate P2X7 receptor and STING, respectively, in neighboring CD8^+^ T cells leading to enhanced type I IFN production-mediated immune activation and tumor suppression, especially at early stage (137).

Numerous studies targeting the STING pathway have been performed to address its potential use as antitumor. Intratumoral delivery of synthetic CDN derivatives induces STING-dependent tumor regression as well as metastases rejection and long-lived immunologic memory in a dose-dependent manner (138, 139). Intravenous CDN administration also increased survival rate in mice with acute myeloid leukemia (140). These results and others indicate potential strong benefit of targeting STING against different types of cancer. While clinical use of immune checkpoint blockade to promote anti-tumor immune responses proved to display tremendous benefit in several types of cancers (141–143), most patients do not show significant improvement when these checkpoint inhibitors are given as a monotherapy and thus require combined chemotherapy (143). Most chemotherapeutic agents non-specifically target dividing cells by blocking DNA replication leading to apoptosis (144). Besides a direct positive effect by killing tumors cells, cytotoxicity and danger signals induced by chemotherapeutic drugs can enhance the inflammatory environment and CD8^+^ T cell activation (145). Therefore, combining immunotherapy with additional targets including the cGAS/STING pathway constitutes an intense scope of investigation, recently reviewed (146). The potential ability of radiotherapy to enhance immunotherapy constitutes another field of investigation. While radiation doses above 12-18 Gy induce DNA exonuclease TREX1 and therefore dampen cGAS activity, repeated irradiation at lower doses (3 times 8 Gy) does not induce TREX1 expression and leads to increased type I IFN production through the cGAS/STING pathway (147). In addition, *Trex1* knockdown restores cytosolic dsDNA accumulation and ISG induction in a mouse model of mammary carcinoma refractory to immune checkpoint inhibitors (147).

In summary, cGAS-STING-elicited outcome in cancer is largely context-dependent. Transient cGAS-STING activation in innate immune cells may display anti-tumor activity, whereas sustained activation might induce immune tolerance and tumor growth. The use of synthetic CDNs together with anti-cancer immunotherapy may be promising in response to certain types of cancers (138, 148).

## Cellular Processes

### STING and Autophagy

Autophagy is a phylogenetically conserved catabolic process induced by numerous endogenous (e.g. nutrient deprivation) or exogenous (e.g. infection) cellular stress conditions aiming to either promote cell survival or apoptosis of senescent cells (149). Autophagy induction by STING has been first identified in the context of bacterial infection. Authors showed that *M. tuberculosis* DNA recognition induces STING-dependent targeting of bacteria and autophagy-mediated resistance to infection (31). More recent studies suggest that STING-dependent autophagy regulation may have evolved before type I IFN induction. In invertebrates such as the sea anemone *Nematostella vectensis*, STING protein does not harbor the two C-terminal tail (CTT) domains critical to activate IRF3 but effectively induces autophagy (30). STING triggers autophagy in a TBK1- and type I IFN-independent manner (32). Upon activation, STING binds to the autophagy inducing protein LC3 promoting both autophagy and STING degradation (32), therefore regulating STING-mediated immune activation. cGAMP binding induces STING translocation to the endoplasmic reticulum-Golgi intermediate compartment (ERGIC) and the Golgi. STING-containing ERGIC serves as a membrane source for LC3 lipidation and autophagasome formation. This form of autophagy, important for the clearance of cytosolic DNA, is mediated by autophagy-related gene 5 (ATG5) and Trp-Asp (W-D) repeat domain phosphoinositide-interacting protein (WIPI2) (30). Furthermore, cGAS protects hepatocytes by triggering autophagy independently of STING in mouse models of ischemia-reperfusion (115).

Cell death following replicative crisis is a senescence-independent process important to prevent oncogenic transformation of pre-cancerous cells with disrupted cell cycle checkpoints. It was shown that cytosolic DNA activates the cGAS/STING pathway to promote macroautophagy and autophagic cell death (150). Of note, STING signaling is negatively regulated by p62/SQSTM1-dependent autophagy pathway activated by TBK1 (151).

Circulating mtDNA levels and STING activation profile are increased in sepsis-induced acute lung injury (ALI) patients (152). In a mouse model of sepsis, mtDNA-triggered STING-mediated IFN production interferes with autophagy by preventing lysosomal acidification and thus worsens pathology (152). STING-dependent autophagy upregulation observed in the striatum of HD patients favors brain damage (127). Radiotherapy-induced mtDNA release facilitates cGAS/STING activation and type I IFN-mediated antitumor responses and autophagy induction limits this effect (153). Patients with breast cancer showing increased genetic autophagy signature in the tumor microenvironment display slightly decreased survival prognostic, inversely correlating with mitochondrial abundance and type I IFN signaling (153).

### CGAS/STING and Cell Death

There is a finely regulated and context-dependent cross-talk between several cell death processes and the cGAS/STING pathway. The latter is often inhibited during apoptotic processes to limit inflammation. During cell intrinsic apoptosis, mitochondrial outer membrane permeabilization (MOMP) leads to mtDNA release but concomitantly to caspase-9 and caspase-3 which display immunosuppressive function by repressing the cGAS/STING/type I IFN pathway (154, 155). It was recently shown that caspase-9 and caspase-3 directly cleave cGAS and IRF3 to limit STING activation and deleterious inflammation (156). In contrast, cGAS-STING pathway initiates certain form of programmed cell death. Nucleosomes competitively inhibit DNA-dependent cGAS activation during regular mitotic processes. However, during mitotic arrest limited cGAS activation leads to mild STING-dependent IRF3 phosphorylation triggering mitotic aberrations and transcription-independent induction of apoptosis (157). In addition, host restriction factor SAMHD1 limits human T cell leukemia virus type 1 infection of monocytes *via* STING-mediated apoptosis and viral products interact with STING to trigger an IRF3-Bax complex leading to apoptosis (158). Noteworthy, T lymphocytes exhibit an intensified STING response predisposing them to apoptosis (34). This effect does not appear to occur in other cell types including dendritic cells and macrophages, presumably owing higher STING expression and signaling in T cells as compared to other cell subsets (34).

Gaidt et al. showed that STING traffics through the Golgi and then through late endosomes and lysosomes (33). The precise location of STING, e.g. at the outer lysosomal membrane or within multivesicular bodies, remains to be determined. STING in lysosomes induces lysosomal membrane destabilization leading to the release of proteases such as cathepsins triggering lysosomal cell death (LCD) (33, 159), however the mechanism involved remains to be established. Furthermore, it appears that the main function of STING targeting to the lysosome is its degradation to limit its activity (160). Necroptosis is a regulated form of necrotic cell death governed by RIP1/RIP3-mediated activation of MLKL. During necroptosis, the proapoptotic BH3-only BCL-2 family member PUMA is transcriptionally activated in an RIP3/MLKL-dependent manner. PUMA promotes the cytosolic release of mtDNA which activates DAI/Zbp1 and STING leading to enhanced RIP3 and MLKL phosphorylation in a positive feedback loop and thus amplifies necroptosis (35). Interestingly, basal cytosolic DNA sensing by the cGAS/STING pathway is important for constitutive type I IFN production and signaling, maintaining baseline ISG induction. It was shown that this phenomenon is critical to reach a critical threshold of MLKL for LPS-dependent necroptosis (161).

### CGAS/STING and Chromosomal Instability/Senescence

Chromosomal instability is a hallmark of cancer in human, associated with poor prognosis, metastasis, and therapeutic failure (129). It results from errors in chromosome segregation during mitosis and can cause micronuclei formation in the cytoplasm. During metazoan cell division, the nuclear envelope is ruptured and if the exposed chromatin is not entirely contained within the daughter cells nuclei, it can be encapsulated within independent structures called micronuclei. Micronuclei rupture leads to cytosolic self-DNA release and cGAS activation (17, 48). DNA damage induced by monogenic autoinflammation (e.g. *Rnaseh2b* deficiency) or exogenous DNA damage (e.g. ionizing radiation) leads to micronuclei formation which rupture activates cGAS leading to ISG expression (17). This pathway is also involved in the context of genotoxic cancer therapy where STING-dependent responses display antitumor activities (48). A recent article showed that chromosomal instability leads to increased numbers of micronuclei and cytosolic DNA, which activate the cGAS-STING pathway (162). Rather than engaging type I IFN or canonical NF-κB pathways, STING activates noncanonical NF-κB signaling linked to the upregulation of epithelial-mesenchymal transition (EMT) and inflammatory genes, therefore enhancing cell migratory capacity and metastasis (162).

cGAS has been shown to be a critical inducer of cellular senescence, a form of terminal cell-cycle arrest associated with pro-inflammatory response which prevents tumorigenesis and participates to the antitumor effects of radio- and chemo-therapies (163). cGAS binds to cytosolic chromatin fragments in senescent cells and induces STING-dependent production of senescence-associated secretory phenotype (SASP) inflammatory mediators promoting paracrine senescence (164). Cytoplasmic chromatin fragments are pinched off intact nuclei during senescence to activate the cGAS-STING pathway (165).

Progeria, or Hutchinson-Gilford progeria syndrome (HGPS), is a rare autosomal dominant condition beginning in childhood with striking phenotypic features of premature aging. It is caused by a truncated lamin A protein (progerin) inducing nuclear envelope fragility and genomic instability and fatal senescence. Progerin-mediated DNA release leads to the upregulation of the cGAS/STING pathway and pathogenic type IFN response (166).

### STING and Inflammasome

Cytosolic DNA-mediated cGAS/STING activation leads to STING trafficking to the lysosome, where it triggers membrane permeabilization and potassium efflux which activate the NLRP3 inflammasome (33). Non-canonical functions of cGAMP in regulating both priming and activation steps of the inflammasome have been reported. cGAS-derived cGAMP induces type I IFN induction *via* STING leading to increased expression of inflammasome components and cGAMP also favors inflammasome activity by promoting complexes containing both NLRP3 and AIM2 (167). Using protein overexpression assays, it was recently shown that Herpes Simplex Virus-1 (HSV-1) infection or cytosolic DNA stimulation triggers STING binding to NLRP3, promoting both NLRP3 localization in the ER and attenuating NLRP3 polyubiquitination and degradation (168). In addition, IL-1β signaling on human myeloid, fibroblast, and epithelial cells induce mtDNA release to activate innate immune signaling *via* cGAS-STING (169) and mtDNA-driven cGAS activation triggers age-related macular degeneration through STING-mediated non-canonical inflammasome pathway involving IFN-β (170).

## Conclusion

Our understanding of the mechanisms of danger signal release and sensing has evolved considerably over the last years. It now becomes clear that misplaced self-DNA is a potent trigger of immune activation through various DNA sensing machinery. Among them, the cGAS/STING pathway has emerged as an important source of type I and type III interferons as well as a critical regulator of cellular processes such as autophagy and programmed cell death. Targeting the STING pathway may offer tremendous therapeutic opportunities, not only in response to infection as recently illustrated with SARS-CoV-2 infection (171, 172) but also in inflammatory conditions and cancer settings. In addition, natural and synthetic CDNs are used as adjuvants to enhance protective humoral and CD4^+^ and CD8^+^ T cells responses in a STING-dependent manner (173–176).

## Author Contributions

Review was written by IC and NR. Figures were prepared by NR. All authors contributed to the article and approved the submitted version.

## Funding

This work was supported by the Centre National de la Recherche Scientifique (CNRS), the University of Orleans, The Region Centre Val de Loire (2003-00085470), the ‘Conseil Général du Loiret’, ‘Fondation pour la Recherche Médicale’ (EQU202003010405) and the European Regional Development Fund (FEDER N° 2016-00110366 and EX005756).

## Conflict of Interest

The authors declare that the research was conducted in the absence of any commercial or financial relationships that could be construed as a potential conflict of interest.

## Publisher’s Note

All claims expressed in this article are solely those of the authors and do not necessarily represent those of their affiliated organizations, or those of the publisher, the editors and the reviewers. Any product that may be evaluated in this article, or claim that may be made by its manufacturer, is not guaranteed or endorsed by the publisher.
